# Emerging Therapeutics in the Fight Against EV‐D68: A Review of Current Strategies

**DOI:** 10.1111/irv.70064

**Published:** 2024-12-19

**Authors:** Nida Kalam, Vinod R. M. T. Balasubramaniam

**Affiliations:** ^1^ Infection and Immunity Research Strength, Jeffrey Cheah School of Medicine and Health Sciences Monash University Malaysia Bandar Sunway Malaysia

**Keywords:** acute flaccid myelitis, broad‐spectrum antiviral, contemporary strains, drug‐resistance, enterovirus‐D68, type‐I interferon, viral life cycle, WIN‐like compounds

## Abstract

Enterovirus‐D68 (EV‐D68) was first identified in 1962 in pediatric patients with acute respiratory conditions in California, USA (US). From the 1970s to 2005, EV‐D68 was underestimated due to limited data and serotyping methods. In 2014, the United States experienced outbreaks of acute flaccid myelitis (AFM) in children EV‐D68 positive. WIN‐like compounds (pleconaril, pocapavir, and vapendavir) bind to the virus capsid and have been tested against various enteroviruses (EVs) in clinical trials. However, these compounds encountered issues with resistance and adverse effects, which impeded their approval by the Food and Drug Administration (FDA). Presently, the medical field lacks FDA‐approved antiviral treatments or vaccines for EV‐D68. Ongoing research efforts are dedicated to identifying viable therapeutics to address EV‐D68 infections. This review explores the current advancements in antiviral therapies and potential therapeutics to mitigate the significant impact of EV‐D68 infection control.

## 1 Introduction

In 1962, enterovirus‐D68 (EV‐D68) was isolated from the pharyngeal swabs of four children with severe acute lower respiratory illnesses in California, USA (US), leading to the identification of the 4 prototype strains, namely Fermon, Rhyne, Franklin, and Robinson. Among these, the Fermon strain is regarded as the prototype of EV‐D68 [1]. EV‐D68 represents a subset of small, non‐enveloped viruses characterized by a positive‐sense single‐stranded RNA genome, classified within the genus Enterovirus of the *Picornaviridae* family [2]. Through methodologies including neutralization assays and sequence analysis of the VP1 major capsid gene, studies have identified 116 serotypes distributed among four specific EV species (A, B, C, and D) that possess the capacity to infect human hosts [2, 3]. EV‐D68 was underestimated from the 1970s to 2005 due to limited data and serotyping methods [4]. Unlike other enteroviruses (EVs) that transmit through fecal‐oral transmission, EV‐D68 spreads through respiratory pathways [5]. EV‐D68 is more similar to rhinoviruses, unstable at 37 °C, and sensitive to acidic environments [6].

Before the summer of 2014, EV‐D68 was mainly linked to respiratory diseases, with rare neurological symptom cases. Between August 2014 and January 2015, EV‐D68 caused an outbreak in the United States that affected 1153 individuals across 49 states and the District of Columbia [7]. Concurrently, there was a surge in acute flaccid myelitis (AFM), a polio‐like illness, with 115 cases reported in 34 states [8]. During the 2014 EV‐D68 outbreak, strains belonging to clade B were the predominant variants identified in circulation [9]. Notably, strains from the lineage of clade B, especially those classified under Clade B1, have been most commonly isolated from patients suffering from AFM [8, 10]. A study found that 12 critical mutations in the B1 clade of EV‐D68 are shared with other paralysis‐causing EVs. Unique to enterovirus‐D70 (EV‐D70) are mutations like 5′UTR/127T and VP2/222T. Poliovirus (PV) exclusively has mutations such as 5′UTR/262C. Enterovirus‐A71 (EV‐A71) is solely characterized by 3D/135S. Some mutations, like VP3/24A, are found in EV‐D70 and PV, while others, like 3D/274K, are present in PV and EV‐A71. Additionally, 5′UTR/188A is common across EV‐D70, PV, and EV‐A71 [8]. Several mechanisms have been hypothesized to elucidate viral dissemination, encompassing the transmission through motor neurons and astrocytes [11]. In a preclinical study, the paralytic strain IL/14‐18952 of EV‐D68 is absorbed by motor neuron axons and transported to the soma via axon microtubules. Within 48 h of intramuscular infection in mice, the viral antigen is detected in the anterior horn of the spinal cord [12]. EV‐D68 has been identified as a cause of AFM in several countries, including the USA [13, 14, 15], Norway [16], Canada [17], and Australia [18]. The number of AFM cases linked to EV‐D68 has been increasing, particularly affecting children. In the United States, respiratory illnesses in children have been associated with this virus [19].

Respiratory illnesses caused by EV‐D68 indicate that the EV‐D68 virus primarily invades the body through the respiratory tract. AFM linked to EV‐D68 amplifies the infection's severity, especially in pediatric populations. The synchronous occurrence of respiratory transmission and virus‐induced AFM constitutes a detrimental interplay, exacerbating health outcomes in vulnerable populations (<5 years old) [10, 13, 20, 21, 22]. Therefore, developing antiviral agents and vaccines is critically essential for the prophylaxis and management of EV‐D68. Currently, no available antiviral treatments or approved vaccines are available to address EV‐D68 infections. This review paper explores the various therapeutic strategies pursued for EV‐D68 and examines their progress in the developmental pipeline.

## 2 Viral Replication Cycle, Transmission, and Clinical Manifestations

**FIGURE 1:**
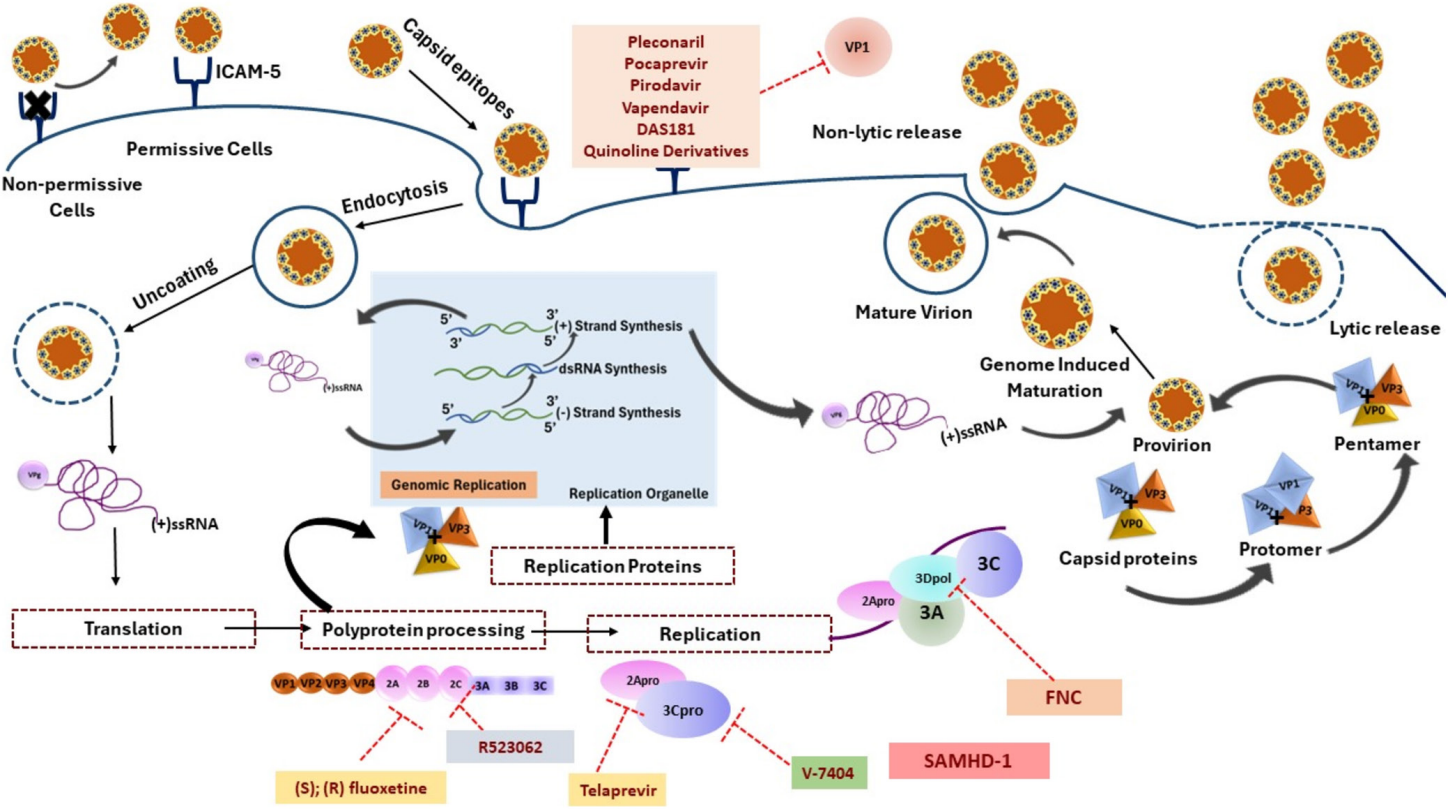
Sialic acid/ICAM‐5 aids the entry of the EVD68 into the host cell. Upon attachment with the receptor, it causes the restructuring of the capsid. It stimulates the formation of intermediate “A particle” that causes perforations in the endosome and aids viral RNA genome delivery. The translation process immediately starts upon entry into the cytosol, and polypeptide processing initiates. The proteolysis of polypeptide separates structural (VP1, VP2, VP3, and VP4) and non‐structural proteins (2A, 2B, 2C, 3A, 3B, 3C, and 3D). Further, the self‐cleavage activity of VP0 leads to the formation of VP2 and VP4 proteins, which further involves capsid maturation. The 3Dpol further proceeds the replication process. The synthesis of (−)ssRNA becomes the source of generating multiple copies of the viral RNA genome. The Vpg‐linked (−)ssRNA and structural proteins help form new matured viral particles. The mature viral particles are released from cytosol either in a non‐lytic or lytic manner.

EV‐D68 is a non‐enveloped, positive‐sense, single‐stranded RNA virus, part of the genus enterovirus within the *Picornaviridae* family. It has a genome of about 7500 nucleotides that encodes a polyprotein cleaved into corresponding viral proteins [23]. EV‐D68 infection cycle starts with capsid attachment to the host cell, facilitating virion concentration at the cell surface. It is followed by entry through receptor‐mediated endocytosis, mediated by an integral membrane protein [20]. Sialic acid (SA) was initially proposed as a receptor facilitating EV‐D68 entry into host cells. A study has confirmed the affinity of EV‐D68 for SA on synthetic glycoproteins. Given its abundant expression in the human respiratory tract, SA is a likely entry point for EV‐D68, suggesting nasal transmission [24]. It was subsequently noted that it only facilitates EV‐D68 entry but is not involved in the conformational changes that occur during viral particle uncoating [24]. Recently, EV‐D68 variants have been reported to enter the host cells without utilizing SA receptors, thus suggesting the presence of alternative receptors for EV‐D68 entry [25]. Intercellular adhesion molecule 5 (ICAM‐5) receptor acts as an entry mediator for both prototype Fermon EV‐D68 and currently circulating EV‐D68 viruses, regardless of their dependency on SA [26]. Recent evidence suggests that ICAM‐5 expression and distribution in mice and neuron models is not required for infection‐induced motor neuron disease. However, these findings raise relevant questions on EV‐D68 entry via ICAM‐5 receptors [12], as shown in Figure 1.

**FIGURE 2:**
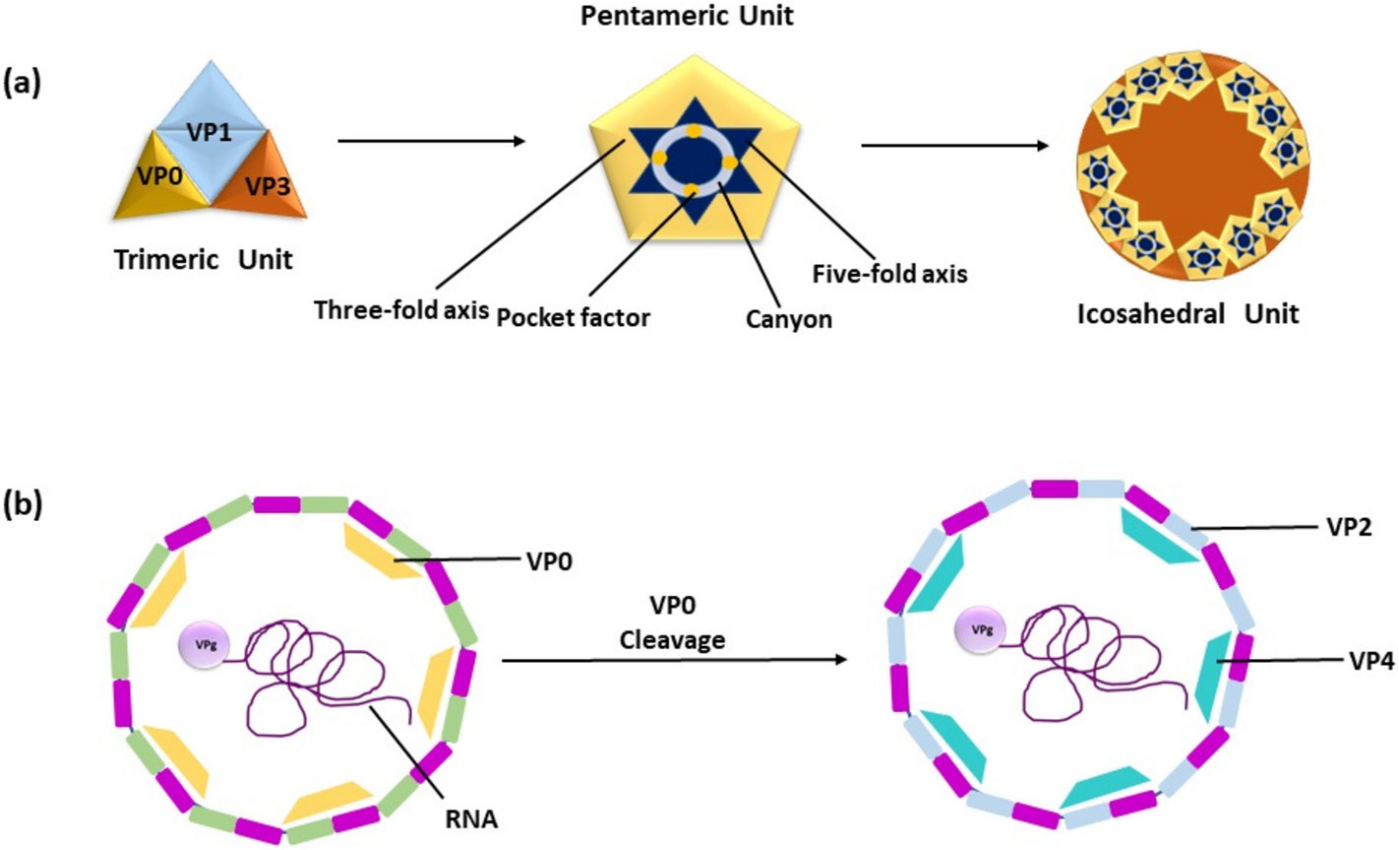
(a) During the replication process, VP0, VP1, and VP3 form a trimeric unit called a protomer. Five units of protomer form pentameric unit, and 12 units of these further form icosahedral capsid. (b) Proteolysis causes cleavage of VP0 protein and forms VP2 and VP4 proteins, which further helps in the assembly of viral particles and capsid maturation.

EV‐D68 adheres to the host cell's surface and gains entry via receptor‐mediated endocytosis. Upon entry, the viral capsid is discarded, liberating its RNA genome into the cytoplasm of the cell. This RNA acts as a template for the synthesis of a large precursor protein, which is subsequently cleaved into functional units, including structural proteins (VP1–VP4) and non‐structural proteins (2A–2C and 3A–3D), facilitated by the proteolytic enzymes of both the host cell and the virus [27, 28]. The virus then undergoes replication, forming a template RNA as a scaffold for producing new viral genomes. This process is localized to specific regions within the cell, known as replication organelles. Assembly of the new virions involves encapsulating the RNA in structural proteins, followed by their maturation within cellular structures termed autophagosomes, which require an acidic milieu to activate, a critical step for the virions to attain infectivity. The egress of the mature virions from the cell is accomplished either through vesicular release or by lysing the host cell [20, 29]. The enteroviral genome encodes a single polypeptide that is proteolytically processed into four structural and seven non‐structural proteins [30]. The enterovirus capsid, characterized by its non‐enveloped icosahedral structure, comprises 60 copies of each of four structural proteins (VP1–VP4). These proteins are organized into subunits, with VP1–VP3 positioned externally and VP4 internally, assembling at vertices to create a pattern of alternating threefold and fivefold symmetry [27, 28]. Adjacent to the pentavalent vertex, a “canyon” is believed to contribute significantly to receptor binding. At the base of the canyon, the hydrophobic pocket in each VP1 subunit houses a host‐derived lipid‐like “pocket factor.” Crystallography research has verified these structural elements in EV‐D68 [24, 28, 31]. The initial cleavage is by a non‐structural protein 2A^pro^, separating structural proteins from the polypeptide. Further processing by non‐structural proteins 3CD^pro^ and 3C^pro^ enzymes generates the structural proteins VP0, VP1, and VP3, along with various non‐structural proteins at different stages. VP2 and VP4 are formed from VP0 during the maturation of the viral capsid [30], as shown in Figure 2. Enterovirus 2B, a transmembrane viroporin, is critical for viral replication [32, 33]. It facilitates intracellular membrane reorganization, creating replication organelles, and enhances virus replication and release by modulating intracellular ion homeostasis, apoptosis, and autophagy [33]. Consequently, targeting 2B in antiviral therapies could effectively impede virus replication [34]. The function of the 2C protein has yet to be entirely elucidated. The roles of the viral 2C protein encompass RNA binding and replication, membrane modification, viral genome encapsidation, and viral progeny assembly [35]. 3C^pro^ of EV‐D68 demonstrates a chymotrypsin‐like fold featuring a catalytic cysteine, histidine, and glutamate triad. Its three‐dimensional structure is similar to the 3Cpro of rhinovirus 2 and poliovirus [36]. The enzyme 3C^pro^ and its precursor form, 3CD^pro^, play a crucial role in producing precursor and mature proteins [36]. The 3D RNA‐dependent RNA polymerase (RdRP), a non‐structural protein of EV‐D68, is essential for viral genome replication, presenting a significant opportunity for drug development [37]. A recent study has observed the impact of EV‐D68 on the cell cycle is facilitated by its non‐structural proteins 3C and 3D. Protein 3C decreases the levels of CDK1 and cyclin B1, leading to the exit from the G2/M phase, while 3D contributes to an increase in cells in the G0/G1 phase by reducing the levels of cyclin D, CDK4, and cyclin E1. These combined mechanisms enable 3C and 3D to effectively promote virus proliferation and infection [23]. Other EVs can be transmitted through the fecal‐oral route; EV‐D68 transmission can occur through the particles that have become aerosolized or through the manual transfer of contaminated substances from environmental surfaces to the airway [38].

In 2014, there was an outbreak of EV‐D68 in the United States that caused respiratory complications in children, such as wheezing, tachypnea, retractions, and breathing issues. This suggests the virus can be transmitted through the respiratory route [39]. EV‐D68 is unique because it shares biological and molecular characteristics of both EVs and rhinoviruses [6]. This makes it especially concerning. EV‐D68 has caused influenza‐like illness (ILI) and AFM in various parts of the world, including the United States [40, 41], Taiwan [42, 43], Europe [44, 45, 46, 47], and Argentina [48]. The respiratory route is highly virulent and dominant for transmitting airborne viruses [49]. Because EV‐D68 can be easily spread through aerosolized droplets and has been associated with AFM, it poses a potential threat to vulnerable populations.

During a mild EV‐D68 infection, the upper respiratory tract (URT) is affected, causing symptoms such as fever and cough. However, a severe infection affects the lower respiratory tract (LRT), leading to wheezing, pneumonia, hypoxia, and difficulty breathing. In rare cases, patients require admission to the intensive care unit (ICU) [50]. Those with a history of asthma or recurrent wheezing are more likely to experience asthma exacerbation [51, 52, 53]. In contrast to poliomyelitis, patients with AFM associated with EV‐D68 predominantly exhibited upper limb involvement, suggesting a predilection for damage to the cervical spinal motor neurons rather than those in the lumbar region. However, there were instances where children presented with extensive limb involvement, encompassing quadriparesis [54, 55]. Some children with severe paralysis require mechanical ventilation due to respiratory failure. Symptoms included facial weakness and swallowing difficulties, indicating brainstem involvement [15, 54]. Magnetic resonance imaging (MRI) findings showed longitudinally extensive T2 and FLAIR hyperintense lesions in the gray matter of the spinal cord, specifically in the anterior horns. Electromyography (EMG) confirmed a denervation pattern consistent with lower motor neuron involvement [56, 57].

## 3 Different Antiviral Strategies Against Non‐Polio Enteroviruses

### 3.1 Host Factors: Prevent Viral Assembly and Replication

Restriction factors are essential to host cellular proteins and are crucial in the primary defense against viral infections. These proteins identify and disrupt certain stages of a virus's replication cycle, effectively preventing the infection. Typically induced by interferon (IFN), restriction factors possess intrinsic characteristics, including their natural expression across various cell types, rendering them autonomously effective [38]. The propagation of human retrovirus‐1 (HIV‐1) and other retroviruses necessitates interactions with proteins in the host cell. Among these host cell proteins, certain restriction factors serve to thwart infection and replication processes [58]. The Sterile Alpha Motif and Histidine‐Aspartate Domain‐Containing Protein 1 (SAMHD1) is identified as one such restriction factor, functioning as a deoxynucleoside triphosphate triphosphohydrolase (dNTPase). This enzyme is pivotal in impeding the infection of HIV‐1 within non‐dividing immune cells, playing a critical role in the mechanism triggered by the host defense in response to the virus [59]. SAMHD1 impedes EV‐A71 replication [60]; nonetheless, the virus has evolved mechanisms to circumvent this blockage. This evasion is achieved by upregulating the host factor TRIM21, which subsequently facilitates the proteasomal degradation of SAMHD1. This study has revealed that the overexpression of TRIM21 correlated with diminished levels of SAMHD1 [60]. A recent study demonstrated that SAMHD1 impedes the function of EVs through competitive interaction with a domain in VP1, which is also responsible for binding with VP2 in EVs. This interaction between SAMHD1 and VP1 hinders the VP1‐VP2 association, ultimately interfering with viral assembly processes [61]. Additionally, this study noted that SAMHD1 exhibits broad‐spectrum antiviral activity against EV‐D68 and other enterovirus, such as EV‐A71 and CV‐A16, independent of its established dNTPase or RNase functions [61].

APOBEC3G (A3G) is a pivotal element in the defense against retroviruses, functioning as a restriction factor that impedes their replication. This is primarily achieved through the hypermutation of the viral complementary DNA and by obstructing the reverse transcription process. A3G exerts its antiviral activities by being incorporated into retroviral capsids, a process facilitated by interacting with the viral genomic RNA during capsid assembly. This strategic positioning allows A3G to effectively deploy its antiviral effects within the capsid, precisely where reverse transcription occurs during the infection phase [62, 63]. 5′ untranslated regions (5′ UTRs) of viral and certain cellular mRNAs, 490 nt sequences termed internal ribosome entry Site (IRES) elements are found in picornaviruses and other viruses like hepatitis‐C virus (HCV) and human immunodeficiency virus (HIV). These elements are classified into four main types:poliovirus (PV), coxsackievirus (CV), rhinovirus (RV), and other EVs. Type I IRES of PV elements generally have five key stem‐loop structures, with the cloverleaf structure at the 5′ end crucial for RNA stability or replication, potentially impacting protein synthesis. The fourth and fifth domains are vital for viral protein production. This study has shown that the cloverleaf and stem‐loop IV in the 5′ UTRs of EVs like PV, EV‐A71, and EV‐D68 bind with the cellular protein PCBP1. Additionally, protein A3G binds to the cloverleaf and stem‐loop II of 5′ UTR of EV‐A71 and EV‐D68, competing with PCBP1 and inhibiting EV replication [64].

Research indicates that EV‐D68 triggers TREM‐1 activation, which plays a role in inflammation by regulating cytokines like IL‐6, IL‐8, and TNF‐α, among others. The activation of TREM‐1 by EV‐D68 depends on NF‐κB p65, as shown by inhibition studies using NF‐κB p65 inhibitors and siRNA. Furthermore, a dual‐luciferase assay revealed NF‐κB p65′s role in binding to the TREM‐1 gene promoter and controlling its expression at the transcription level. Blocking the TREM‐1 pathway with an LP17 inhibitor also reduced the activation of the p38 MAPK pathway, indicating the significant role of TREM‐1 in activating p38 MAPK through phosphorylation [65].

### 3.2 Compounds Targeting Viral Proteins Associated With Viral Attachment and Entry

The VP1 hydrophobic pocket of the capsid protein plays a crucial role in viral attachment, entry, and uncoating. As a result, it is considered to be a suitable target for drug development [66]. The VP1 hydrophobic pocket is engaged with the pocket factor, and various antiviral drugs have been identified targeting this pocket. The capsid binders establish an antiviral state against the EVs by forming interaction with the hydrophobic pocket and displacing pocket factor, which further causes rigidity in the capsid that prevents conformational changes of capsid protein and, as a result, prevents viral protein interaction with the host [66, 67]. Pleconaril, pocapavir, pirodavir, and vapendavir are WIN‐like compounds occupying the hydrophobic pocket in the canyon of VP1, as shown in Table 1 [84, 86]. Pleconaril is a classical drug specifically designed to target the viral capsid of EVs, thereby preventing viral attachment and uncoating viral RNA [86].A study noted that the structure of EV‐D68 closely resembles that of human rhinoviruses (HRVs), against which pleconaril is effective. Pleconaril replaces the pocket factor in the virus, inhibiting EV‐D68 with (EC_50_) value of 430 nM. The pocket factor's size and location in the VP1 pocket match those in HRVs but differ from poliovirus 1 and EV‐A71, making pleconaril more effective against viruses with shorter natural pocket factors, like HRVs and EV‐D68. Analysis of 188 EV‐D68 strains from 1962 to 2013 showed that the VP1 residues interacting with pleconaril are highly conserved, suggesting pleconaril could inhibit many EV‐D68 strains besides the prototype [31]. The clinical development of pleconaril was discontinued in 2002 due to varying outcomes in treating enterovirus‐related infections and its unclear clinical effectiveness against human rhinovirus‐related common colds. Furthermore, pleconaril has been linked to various side effects [87].

**TABLE 1:** Summary of different therapeutic strategies against non‐polio enteroviruses.

Compound name/host factor	Target (viral‐ protein)	Type of study	Enterovirus	Critical findings	Reference
Synthetic drugs target structural and non‐structural proteins					
Isoxazole‐3‐carboxamide analogs of pleconaril (11526092); Pleconaril	VP1	in vitro and in vivo	EV‐D68	11,526,092 (EC_50_ = 59 nM); Pleconaril (EC_50_ = 430 nM); 11,526,092 are well‐tolerated in mice. Both drugs destabilize the capsid of the MO strain; contrastingly, pleconaril causes protein destabilization of the EV‐D68‐Fermon strain. This study signifies distinct mechanisms imposed by drugs with different EV‐D68 strains	[68]
Pleconaril analogs with substitutions at isoxazole and phenyl ring	VP1	in vitro	Pleconaril‐resistant enteroviruses	efficiently inhibited pleconaril‐susceptible enteroviruses within the micromolar range (IC_50_ = 0.02–5.25 μM)	[69]
Pleconaril analogs with substitutions at isoxazole and phenyl ring	VP1	in vitro	Pleconaril‐resistant EVs	combination either with nucleotide polymerase inhibitor or 2C inhibitor delayed the capsid binder‐induced resistance development.	[70]
Quinoline derivatives	VP1	in vitro	EV‐D68	Quinoline derivatives with strong efficacy against EV‐D68, focusing on a key derivative enhanced by an oxadiazole modification for increased potency. Through structure–activity relationship analysis and exploration of the EV‐D68 VP1 protein's structure, compounds showing significant in vitro antiviral activity against various EV‐D68 strains were discovered. Compound 19 showed promising in vitro effectiveness, bioavailability, and metabolic stability, indicating its potential as a novel antiviral agent.	[71]
Compound‐17	Hydrophobic pocket	in vitro	EV‐B, EV‐C, EV‐D, and rhinovirus A and B	Compound 17 and related molecules selectively inhibit the replication of CVB3 by targeting a new pocket on the capsid surface, which is crucial for the conformational changes necessary to release RNA from the virus particle.	[38]
Telaprevir	2A^pro^	in vivo	EV‐D68	Telaprevir during EV‐D68 infection enhances outcomes in AFM in a murine model by significantly reducing apoptosis and viral titers at early time points. Furthermore, telaprevir provided neuroprotective effects on motor neurons and resulted in improved limb paralysis outcomes, extending beyond the site of viral inoculation.	[72]
R523062	2C^Pro^	in vitro	EV‐D68	R523062, a 2C inhibitor, is highly effective against contemporary strains of EV‐D68.	[73]
V‐7404	3C^pro^	Clinical	Multiple strains of EV‐D68	V‐7404 demonstrated a favorable safety and pharmacokinetic profile, warranting further study in treating severe EV infections.	[74]
Rupintrivir	2C^Pro^	in vitro	EV‐D68	Rupintrivir, a protease inhibitor, successfully suppressed the replication of 10 EV‐D68 isolates in vitro, demonstrating mean EC50 ranging from 0.0018 to 0.0030 μM.	[75]
FNC	3D^pol^	in vitro and in vivo	EV‐A71 and CV‐A16	FNC inhibited EV‐A71 EC_50_ = 16.87 nM, indicating potent antiviral activity, as evidenced by dose‐dependent reductions in viral titers in EV‐A71 infected RD cells; FNC demonstrated efficacy in inhibiting the replication of a broad spectrum of viruses, including CV‐A16, CV‐A6, CV‐B3, and EV‐D68.	[76]
Dibucaine Analogs 10a, 12a, 12c	2C^Pro^	in vitro	EV‐D68	A detailed structure–activity relationship led to the synthesis and evaluation of 60 antiviral compounds against EV‐D68 using a cytopathic effect assay. Notably, compounds 10a, 12a, and 12c demonstrated high potency (EC50 < 1 μM) and superior selectivity index (>180) compared to dibucaine across five EV‐D68 strains.	[77]
Quinoline Analog (6aw)	2C^Pro^	in vitro	EV‐D68, EV‐A71 CV‐B3	6aw compound demonstrated broad antiviral activity. EV‐D68 (EC_50_ = 0.2 ± 0.1 μM); EV‐A71 (EC_50_ = 3.1 ± 1.0 μM); and CV‐B3 (EC_50_ = 0.2 ± 0.2 μM).	
Human host factor					
APOBEC3G	5′UTR	in vitro	EV‐A71, CV‐A16, EV‐D68	APOBEC3G (A3G) effectively inhibits various viruses, including EV‐A71 and CV‐A16, by competitively binding with PCBP1 crucial for enterovirus replication. The antiviral activity by A3G extends to EV‐D68 due to similar mechanisms, though it does not affect CV‐A6, highlighting A3G's selective interaction with the 5′UTR as key to its inhibitory effect.	[64]
TREM‐1	NA	in vitro	EV‐D68	EV‐D68‐induced upregulation of TREM‐1 was observed. Furthermore, blocking the TREM1 pathway using LP17, a specific inhibitor, reduced the activation of the p38 MAPK signaling cascade. This indicates that TREM‐1 primarily facilitates the phosphorylation of p38 MAPK. Notably, LP17 treatment, which inhibits TREM‐1, also suppressed viral replication and infection.	[65]
SAMHD1	Interfering interaction between VP1 and VP2	in vitro	EV‐D68	SAMHD1 was found to engage competitively with the domain in VP1, which is also responsible for binding to VP2 in both EV‐A71 and EV‐D68. This action disrupts the interaction between VP1 and VP2, consequently hindering the assembly of the virus.	[61]
TRIM‐25	Overexpression of TRIM‐25 restored RIG‐I expression and IFN‐*β* production	in vitro	EV‐D68 EV‐A71 CV‐A6	Upregulation of TRIM‐25 induced IFN‐based innate immune response against multiple non‐polio EVs	[78]
Immunoglobulin, monoclonal antibodies, interferons, and oligonucleotides					
hIVIG	Neutralization antibodies	in vivo	EV‐D68	hIVIG reduced paralysis and viral load in EV‐D68‐infected mice	[79]
EV‐D68–228; EV‐D68–159	Viral neutralization	in vivo	EV‐D68	228 and 159 efficiently bound at three and five‐fold axes viral particles, respectively; 228 protected animals from respiratory distress and neurological disease	[80]
TRIM‐25	Overexpression of TRIM‐25 restored RIG‐I expression and IFN‐*β* production	in vitro	EV‐D68 EV‐A71 CV‐A6	Upregulation of TRIM‐25 induced IFN‐based innate immune response against multiple non‐polio EVs	[78]
Natural compounds					
Pseudolaric acid B	Cell cycle arrest G2/M	in vitro	EV‐D68	cell cycle arrest at G2/M inhibited EV‐D68 replication	[81]
Andrographolide	Viral RNA and VP1 reduction	in vitro	EV‐D68	ADO significantly reduced viral RNA and VP1 protein production levels in infected cells. This study highlighted that ADO does not interfere with the 5′ untranslated region (5′ UTR), which is essential for viral RNA replication and protein synthesis in enteroviruses. This suggests ADO acts before viral replication and protein synthesis initiates.	[82]
Avoenin	Uncoating and VP1	in vitro	EV‐D68	Avoenin tested against the EV‐D68 virus, avoenin showed an EC_50_ of 2.0 μM, making it about ten times less effective than the known drug pleconaril, which has an EC_50_ of 0.23 μM. The research highlighted avoenin's unique structure and action mechanism, which targets the viral uncoating process in EV‐D68 infections. Although avoenin's potency is not as high as other enterovirus inhibitors that work in the nanomolar range, its discovery in avocado marks it as a noteworthy substance with significant activity against EV‐D68.	[83]
Enzyme/repurposed drugs					
DAS181	Cleaves sialic acid present on the cell surface	in vitro	EV‐D68	efficiently inhibited both historical and contemporary strains.	[84]
Fluoxetine	NA	Cohort	EV‐D68induced AFM	Cases of AFM treated with fluoxetine were more frequently associated with EV‐D68 infection, suggesting clinicians preferably use fluoxetine for suspected or confirmed EV‐D68 cases. This link between fluoxetine treatment and higher EV‐D68 prevalence may indicate that EV‐D68 infections lead to more severe AFM symptoms.	[85]

Abbreviations: 5′UTR, 5′ untranslated region; CPE, cytopathic effect; FNC, 2′‐deoxy‐2′‐*β*‐fluoro‐4′‐azidocytidine; PCBP1, poly(C)‐binding protein 1.

Pocapavir (V‐073) is an antiviral agent designed to target the capsid of poliovirus (PV), preventing viral entry into the host cell [88]. Pocapavir demonstrated significant antiviral potential in pre‐clinical studies against PV [89, 90]. Human studies on pocapavir confirmed its safety and efficacy in enhancing viral clearance. However, treatment was associated with resistance and increased viral transmission [88].

Vapendavir, a capsid inhibitor, significantly improved asthma exacerbation caused by HRV [66]. However, a study has shown the effectiveness of two capsid inhibitors, including pocapavir and vapendavir, revealed their lack of efficacy against three strains of EV‐D68 identified during the 2014 outbreak (MO/18947, USA‐MO/18949 and USA‐IL/18956). These findings extend to the prototype strain, Fermon (1962), indicating a broader resistance of EV‐D68 to these treatment options [84]. Similarly, a recent study identified a tetrazole‐based compound, R856932, as a potential novel antiviral agent. It interacts with the VP1 protein of the viral capsid, inhibiting the viral uncoating process and preventing viral genome release into host cells. This compound showed significant efficacy against various EV‐D68 strains, particularly strain US/MO/14–18947, with an EC_50_ of 0.46 μM and a CC_50_ of 32.0 μM. Its broad‐spectrum antiviral activity was confirmed against four contemporary EV‐D68 strains (US/MO/14–18949, US/MO/14–18947, NR‐49129, US/IL/14–18952, US/KY/14–18953, and US/IL/14–18956), with EC_50_ values ranging from 1.40 to 4.36 μM. Resistance to R856932 emerged via capsid proteins VP1 and VP2 mutations, specifically VP1‐A129V and VP2‐T139A. These mutations affect the binding of the compound, with VP1‐A129V directly impacting interaction and VP2‐T139A altering drug entry. Molecular docking and reverse genetics analyses revealed an overlapping binding site between R856932 and pleconaril, highlighting the compound's mechanism and potential resistance pathways [91].

A study explored novel quinoline derivatives, identifying several with strong efficacy against EV‐D68. A key derivative featured an oxadiazole modification, enhancing its potency through structural optimization. Analysis of the structure–activity relationship (SAR) and the VP1 protein's structural biology led to identifying compounds with significant in vitro antiviral activity, including broad‐spectrum efficacy against various EV‐D68 strains (prototype and contemporary). Quinoline analogs were developed as anti‐EV‐D68 agents using structure‐based virtual screening and SAR. The leading compound, 19, demonstrated superior broad‐spectrum antiviral activity attributed to the 1,2,4‐oxadiazole group facilitating binding to EV‐D68 VP1's hydrophobic pocket. Compound 19's in vitro effectiveness and promising bioavailability and metabolic stability in rats and human liver microsomes suggest its potential as a novel antiviral agent [71].

A recent study has identified a new drug target for coxsackie‐B3 (CV‐B3), a region located at the capsid interface called conserved VP1–VP3 interprotomer. The study also found several sites in the pocket that could not be mutated, emphasizing the importance of the pocket in the replication of various enterovirus species. Moreover, antiviral studies of compound‐17 and its analogs have shown significant potential against EV‐B, EV‐C, EV‐D, and rhinovirus A and B [38]. A protein called DAS181 is fused with sialidase, which helps it capture and remove SA from the surface of cells. This activity prevents viruses from attaching and entering the cells. DAS181 is highly effective in fighting against pandemic strain H1N1, avian flu, and drug‐resistant influenza viruses [92, 93]. Recently, DAS181 reduced oxygen demand in parainfluenza virus (PIV) induced hypoxia in patients [94]. An in vitro study has shown that DAS181 significantly inhibited contemporary strains (USA‐MO/18947, USA‐MO/18949, and USA‐MO/18956) and the prototype strain of EV‐D68 (Fermon) [84].

### 3.3 Compounds Target Viral Proteins Associated With Replication

A recent study indicates that the 2C protein of EV‐D68 plays a key role in viral‐host interactions, facilitating infection. Fluoxetine, a serotonin reuptake inhibitor, disrupts EV‐D68 replication by targeting the 2C protein, highlighting a potential therapeutic strategy [95]. Interestingly, a recent study showed that (S)‐fluoxetine interacts with 2C to hinder the replication potential of CV‐B3 at a 5‐fold lower EC_50_ compared to racemic (R)‐fluoxetine [96], as shown in Table 1.

A recent finding illuminates alternative action modes against EVs, potentially minimizing drug‐related side effects. Four non‐chiral, tri‐fluoro compounds were evaluated for broad‐spectrum antiviral efficacy against emergent enterovirus strains, echoing fluoxetine's structure [97]. The experimental data indicate that new analogs exhibit broad antiviral activity through interaction with the 2C viral protein without the neuroactivity associated with fluoxetine. In pre‐clinical trials, these analogs require established safety profiles before approval [98]. Previously, rupintrivir was an effective protease inhibitor against EV‐D68 [99], EV‐A71 [100], and HRV‐87 [74]. Interestingly, rupintrivir and V‐7404 showed high inhibition potential against historic strain and recently emerged strain, with EC_50_ values ranging from 0.0015–0.005 μM ^69^. V‐7404, a 3C^pro^ inhibitor of poliovirus, oral administration of V‐7404 across all cohorts demonstrated effective absorption without notable accumulation. Pharmacokinetic (PK) exposure escalated in a dose‐proportional manner, showing no dependency on time. V‐7404 was generally well‐tolerated, presenting a favorable safety and PK profile, thus endorsing its continued clinical evaluation for serious infection caused by EVs [74]. The 2A protease of the EV‐D68 strain US/KY/14–18,953 has shown neurotropism and was linked to paralysis in children during the 2014 US outbreak. The antiviral efficacy of telaprevir was assessed in RD cells infected with this strain, revealing specific cleavage at the VP1‐2A junction of the EV‐D68 polyprotein. Telaprevir displayed low cytotoxicity in RD cells (CC50 = 46.2 ± 14.3 μM, selectivity index = 77) over 3 days. Since the 2A protease is conserved across EV‐D68 strains, telaprevir is expected to be effective against other strains [101].

Azvudine (FNC), a novel cytidine analog, demonstrates a higher affinity for deoxycytidine kinase, exhibiting phosphorylation rates up to three times higher than deoxycytidine. A study assessed the antiviral efficacy of FNC against multiple EVs, such as EV‐D68, CV‐B3, CV‐A6, and CV‐A16, with EC_50_ values of 1.58, 33.78, 13.43, and 52.12 nM, respectively.

In addition, this study also evaluated seven analogs of FNC. This study noted that only FNC significantly reduces viral VP1 protein, RNA levels, and titers in vitro, indicating its potential to block viral RNA synthesis and suggesting its viability as a therapeutic against infection caused by multiple EVs [76].

### 3.4 Drug Resistance and Combination Therapy for the Treatment of Enterovirus Infection

Pleconaril was initially developed to address the common cold caused by human rhinoviruses and even reached Phase III of clinical trials. However, the FDA declined to approve it for this purpose, citing concerns over side effects and the potential for it to induce drug‐resistant mutations [87]. It was identified that the pleconaril‐resistant hRV2 isolates possess the I99F mutation within the binding pocket [102]. Similarly, the V69A mutation found in a pleconaril‐resistant variant of EV‐D68 is also located in this pocket [75]. The pocket mutation A156T observed in the vapendavir‐resistant isolate of EV‐D68 aligns with the A150 T/V mutation previously documented in the pleconaril‐resistant hRV14 [102]. We observed mutations in hRV2 and EV‐D68 outside the drug‐binding pocket. The hRV2‐resistant isolate exhibited a vapendavir‐dependent phenotype, requiring the drug for infectivity and showing impaired replication without it. In contrast, resistant EV‐D68 variants with the K167E mutation in the VP1 protein loop could replicate independently of vapendavir, resembling isolates with binding pocket mutations. The mutations in pockets A156T and M252L likely drive the EV‐D68 resistant phenotype, as the glutamic acid at position K167E in the CU70 strain (K155 in the Fermon prototype) is naturally found in various EV‐D68 strains that capsid binders can inhibit. Notably, this amino acid is also present in strain 4310901348, which is susceptible to pleconaril and pirodavir but resistant to vapendavir [75].

To tackle issues related to drug resistance and toxicity, as well as to effectively target various enteroviruses, antiviral drugs can be utilized in combination therapies [103].

Several combinations of two or three drugs, including vapendavir, pleconaril, rupintrivir, and enviroxime, have been evaluated both in vitro and in vivo against multiple viruses such as EV‐A71, PV, CV‐B1, CV‐B3, HRV‐A2, and HRV‐B14 [104, 105, 106, 107].

A study has explored the potential of the triple‐drug combination of pleconaril, rupintrivir, and remdesivir and demonstrated significant efficacy against echovirus (EV) 1, EV6, EV11, and coxsackievirus B5 in human lung epithelial cells. The triple cocktail outperformed monotherapy and dual‐drug therapy, effectively protecting cells from EV1‐induced cytotoxicity over seven passages. Additionally, it exhibited strong antiviral activity against EV‐A71 in human intestinal organoids. This study highlighted that the oral administration of pleconaril and its analogs, along with rupintrivir and remdesivir, highlights the potential for further pre‐clinical development of this combination. These agents target the viral capsid protein VP1 and inhibit viral 3C protease and 3D polymerase. Such combinations could effectively suppress multiple picornaviruses [108].

### 3.5 Challenges in Therapeutic Development for the Treatment of EV‐D68 Infection

There are no approved therapies for EV‐D68, although several compounds have shown in vitro efficacy, including Rupintrivir, Enviroxime, and Pleconaril [109]. Established therapies demonstrating efficacy for EV‐D68 are currently lacking, especially for complications like AFM. The conduct of randomized controlled trials to investigate potential treatments for this condition is complicated by its rarity and the erratic geographic and temporal distribution of cases. Appropriate in vitro and in vivo models may facilitate the rational development of empirical therapies [79].

A significant reason for the absence of approved treatments is the need for a suitable animal model for researching experimental therapeutics against EV‐D68 until recently. A cotton rat infection model with non‐adapted EV‐D68 was established by Patel et al. (2016) [110]. Furthermore, a model demonstrating the neurotropic effects of EV‐D68 in neonatal Swiss Webster mouse pups was reported by Hixon et al. (2017) [4]. Notably, developing a neonatal mouse model displaying EV–D68–induced paralysis has proven beneficial for assessing potential treatments for AFM [79]. A recent study reported a respiratory disease model for EV‐D68 infection in mice, demonstrating that a mouse‐adapted virus effectively infects 4‐week‐old AG129 mice. The model illustrates rapid viral replication in the lungs, with subsequent spread to the bloodstream and other tissues, reflecting the acute nature of human infections. Additionally, the model utilizes the natural infection route and facilitates antiviral therapy evaluation, contrasting with other models that require more complex procedures or are cost‐prohibitive [109]. Notably, developing a neonatal mouse model displaying EV–D68–induced paralysis has proven beneficial for assessing potential treatments for AFM [79].

A recent study explored the structure–activity relationships of a compound targeting the 2C protein of EV‐D68, EV‐A71, and coxsackievirus (CV)‐A24v. This compound, structurally similar to the FDA‐approved fluoxetine, exhibits better chemical properties. Several novel inhibitors with broad‐spectrum efficacy against EV and RV were identified, revealing a shared druggable binding site on their 2C proteins. The synthesized analogs resemble fluoxetine but lack the tri‐fluoro group essential for SSRI activity, necessitating further in vivo validation [97]. A recent study identified novel quinoline analogs as potential anti‐EV‐D68 agents through structure‐based virtual screening and rational drug design. Compound 19 exhibited broad‐spectrum antiviral activity against multiple EV‐D68 strains, surpassing pleconaril. A key feature of its efficacy was the 1,2,4‐oxadiazole substituent, which facilitated binding to the hydrophobic pocket of the EV‐D68 VP1 capsid protein. In‐vitro studies showed compound 19 effectively inhibited viral protein expression and replication, primarily through its interaction with VP1. Additionally, it demonstrated good bioavailability in rats and metabolic stability in human liver microsomes, indicating a need for further investigation [71].

TDD uses protein structures from crystallography and homology modeling to explore drug design. Structural data aids in discovering new drugs via practical design virtual screening and optimizing existing ligands. Understanding differences between wild‐type proteins and drug‐resistant mutants can help specifically target the latter [111].

Modeling the AFM phenotype resulting from respiratory EV‐D68 infection presents significant challenges due to the infrequent occurrence of paralytic disease in humans. Even under optimal conditions, detecting AFM necessitates large cohorts of animals, given the rarity of these infections. To enhance disease incidence in mouse models, researchers have employed neonatal subjects, immunodeficient strains, and mouse‐adapted virus variants, resulting in successful viral replication. However, the absence of standardized methodologies across existing studies complicates comparisons and interpretations. Ongoing refinement and the harmonization of these models are crucial for the efficacious screening of potential treatments for EV‐D68 [112].

In conclusion, while progress has been made in understanding and modeling EV‐D68 infections, significant challenges remain in developing effective therapies, particularly for associated complications like AFM. Establishing appropriate animal models has been crucial for advancing research, yet the rarity and variability of cases complicate clinical trial efforts. Continued innovation in drug design and standardization of experimental methodologies will be essential for identifying viable treatments and enhancing our ability to combat this viral infection.

### 3.6 Immunoglobulin, Monoclonal Antibodies, Interferons and Oligonucleotides

Human intravenous immunoglobulin (hIVIG) comprises polyspecific IgG isolated from healthy donors' plasma [113]. Zhang et al. found that commercial IVIG has high titers (9.5log_2_ to 17.5log_2_) of neutralizing antibodies against EV‐D68, indicating shared antigenic sites with prototype strains and unique sites distinct from historical strains [114]. Interestingly, hIVIG reduced the incidence of paralysis onset and decreased the motor impairment score in the treated group more than in the infected control group [79]. The use of IVIG has proven beneficial in neonates severely infected with enteroviral diseases [79]. Administration of IVIG to infected patients rapidly decreased viral load due to pathogen‐specific neutralizing antibodies [115, 116].

In 1986, the US FDA approved the first monoclonal antibody (mAb), OKT3, for preventing solid organ rejection in transplant recipients [117]. mAbs protect against viral infection by binding to free viral particles and causing viral neutralization [118]. Numerous mAbs have already been approved by the US FDA for treating different diseases [118]. Recent cryo‐electron microscopy studies demonstrate that EV‐D68‐228 binds to the viral capsid's five‐fold axes. Notably, pre‐ or post‐infection administration of EV‐D68‐228 offers protection against neurological and respiratory diseases in mice [80]. Similarly, intranasal administration of monoclonal antibody A61 effectively neutralizes EV‐D68 in neonatal mice by binding to the VP1's DE loop, disrupting virus‐cell interaction via *α*2,6‐linked sialic acids [119]. Recent studies have identified two monoclonal antibodies, 15C5 and 11G1, with unique neutralization mechanisms against EV‐D68. 15C5 targets the three‐fold axis, inducing mature viral particles to transition into A‐particles and mimic ICAM‐5 receptor interaction. Conversely, 11G1 binds to the five‐fold axis, facilitating A‐particle recognition [120].

The innate immune system serves as the initial barrier against viral infections. Upon virus detection, host cells initiate signaling pathways that produce interferon proteins and activate genes stimulated by interferon, which have antiviral effects [121]. Interferon production and signaling are commonly believed to follow a two‐phase amplification process. Initially, IFN‐*β* is produced by virus‐infected cells in an IRF‐3‐dependent manner, stimulating the expression of interferon‐stimulated genes (ISGs), including IRF‐7. The activation of IRF‐7 leads to further production of IFN‐α and IFN‐*β* through a positive feedback mechanism [122]. Recognition of viral RNA components by pattern recognition receptors (PRRs), such as TLR3, TLR7, TLR8, and RIG‐I, induces type‐I interferons that develop an innate immune response against the pathogen [123]. Therefore, interferons are critical in establishing innate immunity and controlling viral replication within mammalian hosts [124]. Yang *et al.* noted that type‐I interferon receptor deficiency lowered the survival rate in CV‐A16‐infected mice [125]. More recently, the critical role of type‐I interferon (IFN) in initiating innate immunity. An enzymatic assay demonstrated the degradation of interferon regulatory factor‐9 (IRF9) by 3C^pro^ EV‐A71, implicating a disruption in the IFN‐signaling pathway [124]. Subsequently, an alliance of IFN‐α and rupintrivir showed high synergy in abolishing the replication of EV‐A71 [125]. Similarly, EV‐D68 targets protease 3C^pro^ encoded in Q167 and Q189. This leads to the degradation of IRF7, which activates innate immunity in response to EV‐D68 [126]. These studies collectively suggest that 3C^pro^‐mediated downregulation of IRFs impairs innate immune responses by disrupting IFN‐signaling pathways [125, 126].

E3 ubiquitin ligase TRIM25, which belongs to the tripartite motif (TRIM) family of proteins, has been identified as a critical component in activating the innate immune response to RNA viruses [127]. The detection of viral pathogens in host cells is mediated by PRRs, which initiate an antiviral response [128]. RIG‐I, a key member of the RIG‐I‐like receptor (RLR) family of PRRs, is found in a dormant state within uninfected cells, with its N‐terminal tandem CARDs (caspase activation and recruitment domains) being inactive for signaling purposes [129]. Upon activation, RIG‐I triggers a signaling pathway that relies on the E3 ligase function of TRIM25, leading to the production of IFNs and inflammatory cytokines [130]. The primary function of TRIM25 within the RIG‐I pathway is highlighted by discoveries indicating that viruses, including influenza A virus (IAV) and dengue virus (DENV), have developed strategies to inhibit RIG‐I signaling by directly targeting and impairing the activity of TRIM25 [131, 132]. Research has shown that the 3C_pro_ proteins of enterovirus can inhibit the RIG‐I signaling pathway. This inhibition occurs by reducing RIG‐I and TRIM25 levels, both crucial for triggering the type I interferon (Type‐I IFN) pathway via RIG‐I. Most proteins within the human TRIM family are identified by possessing at least four specific domains: an N‐terminal RING domain, a central coiled‐coil domain, a SPRY domain, and one or two B‐boxes. These structural domains involve various biological functions, including cellular activities, and play a significant role in antiviral and antimicrobial defenses [78]. This study shows that overexpression of TRIM25 was linked to restoring RIG‐I expression and IFN‐*β* production, leading to an innate immune response against several EVs, including EV‐D68, CV‐A6, and EV‐A71. However, this response was not observed against CV‐A16 [78].

### 3.7 Natural Compounds With Anti‐Nonpolio Enteroviruses

At present, numerous herbal compounds and their bioactive metabolites have become a significant focus of research due to their efficacy and cost‐effectiveness [133]. Traditional Chinese Herbal Medicine (TCHM) is widely used to eradicate infections related to viruses [134]. However, systematic methodologies have been developed to explain the antiviral mechanism imposed by bioactive metabolites [134]. Pseudolaric Acid B (PB), a compound extracted from *Pseudolarix kaempferi* Gordon, is predominantly utilized in managing dermatological conditions [135]. PB mitigated skin lesions and improved severity scores in atopic dermatitis mice by reducing pro‐inflammatory cytokines, inflammatory cell infiltration, and serum IgE levels [135]. Research has shown that the pseudolaric acid B markedly decreases the amounts of viral RNA and the synthesis of EVD68 (US/KY/14‐18953) compared to the control cohort. The disparity in TCID50/ml and mRNA quantities between the cohorts administered with the control agent and pseudolaric acid B was 234 and 2.33‐fold, respectively. This implies that pseudolaric acid B predominantly influences the final phase of viral maturation but also interferes with viral gene replication [81]. Various other Chinese medicinal herbs, including artemisinin, rheum emodin, astragaloside, erianin, and oridonin, were tested against EV‐D68 for their antiviral efficacy. However, Pseudolaric acid B significantly reduced viral replication and protected cells from cytopathic effects. This was attributed to its ability to induce G2/M phase arrest in the host cell cycle, a characteristic shared with oridonin and erianin, which also showed reduced viral replication. These findings suggest compounds inducing G2/M phase arrest, like pseudolaric acid B, oridonin, and erianin, could be promising in treating EVD68‐related diseases, highlighting the need for further investigation into Chinese medicinal herbs for potential therapeutic applications [81].

*Andrographis paniculata* has been an important part of traditional herbal medicine in Asia [136] and Scandinavia for a long time [137]. Recent research demonstrated the significance of its main active component, andrographolide (ADO), and its antiviral, anti‐parasitic, antimicrobial, and anti‐inflammatory properties. Studies have shown that ADO effectively treats respiratory infections caused by the influenza A virus (IAV) by inhibiting the IAV‐induced RIG‐I‐like receptor signaling pathway in human bronchial epithelial cells [138]. A study evaluated the antiviral potential of ADO against the EV‐D68 prototype strain (Fermon) and its variants from the 2014 US outbreak (US/KY/14‐18953, US/MO/14‐18947, and US/KY/14‐18953) by using RD cell cultures. During the infection process, it was noted that EV‐D68 initiates RNA replication and VP1 protein production. ADO significantly lowered intracellular viral RNA levels and reduced VP1 protein production. The function of the 5′ untranslated region (5′ UTR) is crucial for protein synthesis and the replication of viral RNA in EVs like poliovirus, EV‐A71, CV‐A16, and rhinovirus. The research indicated that ADO's mode of action does not disrupt the 5′ UTR function of EV‐D68 in tests assessing the viral RNA polymerase 3D's dependence on 5′ UTR reporter activity. This points to ADO acting at a stage before initiating viral RNA replication and protein synthesis [82].

A study isolated and characterized a novel molecule, 2R,4R‐(12Z,15Z)‐heneicosa‐12,15‐diene‐1,2,4‐triol, avoenin, from *Persea americana* (avocado). The efficacy of avoenin against prototype EV‐D68 was evaluated, revealing an EC_50_ of 2.0 μM, approximately ten times less potent than the capsid inhibitor pleconaril, which has an EC_50_ of 0.23 μM. Furthermore, this study noted the avoenin's structure and demonstrated its mechanism of action, which involves targeting the uncoating process of EV‐D68 infection. Despite the EC_50_ of avoenin not being particularly low compared to other capsid‐targeting enterovirus inhibitors operating in the nanomolar range, the study signifies the identification of avoenin from avocado as a compound with considerable anti‐EV‐D68 activity [83].

## 4 Conclusion

The resurgence of EV‐D68, particularly its association with AFM in children, underscores an urgent need for effective antiviral and vaccine strategies against virulent strains that have emerged recently. However, Pleconaril and other capsid inhibitors failed to show potent antiviral activity against other EVs and developed resistance and unwanted side effects in the clinical setting; therefore, they could not attain approval from the FDA. Despite their failure in clinical trials against other EVs [87], pleconaril and other capsid binders were evaluated for their antiviral potential against both historical and contemporary strains of EV‐D68 [31].

More recently, novel quinoline derivatives, mainly featuring an oxadiazole modification, for their potent antiviral activity against EV‐D68. The leading compound, 19, distinguished itself through its broad‐spectrum efficacy and strong in vitro performance, attributed to its enhanced interaction with the EV‐D68 VP1 protein. Further research into these compounds' bioavailability and metabolic stability could pave the way for new therapeutic strategies against EV‐D68 infections [71]. Protease inhibitors (PI) have been evaluated against multiple EV‐D68 strains. Rupintrivir was found to be effective against 10 strains of EV‐D68 strains. However, rupintrivir‐resistant mutation, characterized by a single amino acid alteration, highlights the complex interplay between viral protein structure and drug efficacy. Although located significantly distant from the inhibitor's binding site, this mutation indirectly affects rupintrivir's binding efficiency through subtle structural modifications rather than direct interactions. These findings underscore the importance of considering both proximal and distal mutations in designing and developing antiviral therapeutics, particularly for those targeting the highly conserved 3C protease across enterovirus species [75].

Currently, no FDA‐approved drugs and vaccines against EV‐D68 are available, but human intravenous immunoglobulin (hIVIG) could be a promising treatment for EV‐D68 infection. High titers of neutralizing antibodies within hIVIG can recognize both shared and unique antigenic sites of EV‐D68 strains. This recognition is crucial in reducing paralysis incidence and decreasing motor impairments, offering significant therapeutic benefits, especially in neonates with severe enteroviral diseases, by rapidly reducing viral loads [79].

Exploring herbal compounds and their active components, particularly those derived from TCHM, has shed light on their potential as effective and affordable treatments against viral infections. Pseudolaric acid B, from Chinese medicinal herbs, and andrographolide from *A. paniculata* , have shown potential in inhibiting viral maturation and replication, particularly in respiratory infections. They act by decreasing viral RNA synthesis and targeting specific signaling pathways. Additionally, avoenin from *P. americana* targets the viral uncoating process, offering a promising avenue against EV‐D68, albeit with a lower efficacy. These findings highlight the need for further research into these compounds as potential treatments for EV‐D68‐related diseases [83, 135, 138].

Restriction factors are host cellular proteins that play a key role in defending against viral infections by targeting and interfering with different stages of a virus's replication cycle. Host factors such as SAMHD1 and A3G are crucial in defending against viral infections like HIV‐1 by disrupting various stages of the viral replication process [59, 62]. SAMHD1 disrupts EV‐D68 assembly by inhibiting VP1 and VP2 protein interaction, essential for viral construction. It is noted that the cloverleaf and stem‐loop IV in the 5′ UTR of enteroviruses like PV, EV‐A71, and EV‐D68 interact with the cellular protein PCBP1. Furthermore, protein A3G competes with PCBP1 by binding to the cloverleaf and stem‐loop II in the 5′ UTR of EV‐A71 and EV‐D68, hindering enterovirus replication [61, 64]. In conclusion, the challenge of combating EV‐D68, particularly given its link to AFM in children, demands innovative and multifaceted therapeutic approaches. Despite setbacks with traditional antivirals such as pleconaril, promising developments in novel quinoline derivatives, protease inhibitors, and the exploration of herbal compounds, alongside the potential use of hIVIG, offer new paths forward. Additionally, the role of host restriction factors in viral defense highlights the importance of a comprehensive understanding of viral‐host interactions in designing effective treatments. Further, combination therapies, such as those incorporating pleconaril, rupintrivir, and remdesivir, show promise in pre‐clinical studies, but more research is needed for clinical application. Establishing suitable animal models for EV‐D68 is crucial for advancing therapeutic development and understanding the virus's neurotropic effects, particularly in AFM. Future research should focus on the rational design of inhibitors and further evaluation of combination therapies, potentially broadening our arsenal against not only EV‐D68 but also other enteroviruses. As we strive for effective treatments, collaborative efforts in clinical trials and drug development will be essential in overcoming the obstacles posed by this viral family.

## Author Contributions

VB conceptualized and designed the manuscript. NK wrote and prepared the manuscript and designed all the figures. VB critically revised the manuscript. All the authors approved the final version.

## Conflicts of Interest

The authors declare no conflicts of interest.

### Peer Review

The peer review history for this article is available at https://www.webofscience.com/api/gateway/wos/peer‐review/10.1111/irv.70064.

## Data Availability

This review article is based on publicly available literature and datasets. All sources used are cited in the references section. No new data were generated or analyzed specifically for this review.
